# Functional inhibition of F11 receptor (F11R/junctional adhesion molecule-A/JAM-A) activity by a F11R-derived peptide in breast cancer and its microenvironment

**DOI:** 10.1007/s10549-019-05471-x

**Published:** 2019-10-24

**Authors:** Radoslaw Bednarek, Anna Selmi, Dagmara Wojkowska, Kamil Karolczak, Marcin Popielarski, Marta Stasiak, Moro O. Salifu, Anna Babinska, Maria Swiatkowska

**Affiliations:** 1 Department of Cytobiology and Proteomics, Medical University of Lodz, Lodz, Poland; 2 Department of Haemostasis and Haemostatic Disorders, Medical University of Lodz, Lodz, Poland; 3 Department of Medicine, State University of New York, Downstate Medical Center, Brooklyn, NY USA

**Keywords:** Breast cancer, JAM-A, Platelet F11 receptor, F11R, Metastasis, Endothelium

## Abstract

**Purpose:**

To examine the involvement of the F11R/JAM-A protein in breast cancer metastasis, we utilized the F11R/JAM-A antagonistic peptide 4D (P4D) in experiments of transendothelial migration (TEM) of breast cancer cells.

**Methods:**

Experiments were conducted in the mouse 4T1 breast cancer model utilizing the human mammary epithelial cell and endothelial cell lines. The levels of soluble F11R/JAM-A (sJAM-A) in the murine plasmas were measured by ELISA. Levels of F11R/JAM-A mRNA and protein in cell lines were assessed by qRT-PCR and Western blot, respectively. Cell surface expression of F11R/JAM-A was demonstrated by flow cytometry. Functional tests included the TEM of breast cancer cells and adhesion of breast cancer cells to the endothelium. The endothelial permeability was studied by fluorescent tracer assay and by the Real-Time Cell Analysis (RTCA).

**Results:**

The tumor inducers Tβ4 and TGF-β1 reduced the levels of sJAM-A in murine plasma, and reduced the F11R/JAM-A protein levels in the human microvascular endothelial cell line HMEC-1. The adhesion and TEM measured between breast cancer cells and inflamed or Tβ4-treated endothelium were inhibited by P4D. The presence of P4D did not destabilize the pre-existing tight junctions in the endothelial monolayer. The barrier-protecting effect of P4D was stronger than that of forskolin, when a booster dose of P4D was applied to the inflamed endothelium.

**Conclusions:**

F11R/JAM-A protein can be considered as a novel target in the treatment of breast cancer metastasis. In vivo and clinical studies are needed to further investigate the effectiveness of F11R/JAM-A-derived peptide as a possible anti-metastatic drug.

**Electronic supplementary material:**

The online version of this article (10.1007/s10549-019-05471-x) contains supplementary material, which is available to authorized users.

## Background

Breast cancer is the leading cause of cancer death among females worldwide, [1] including Poland [2]. The majority of breast cancer-related deaths is caused by metastasis that is considerably promoted by inflammation [3]. The formation of metastasis is initiated by the adhesion of tumor cells to the endothelium and the subsequent transendothelial migration (TEM) of cancer cells [4]. This mechanism is not completely understood. Hence, we focused on the molecule involved in the adhesion of platelets to cytokine-inflamed endothelium, the F11 receptor (F11R, Junctional Adhesion Molecule-A, F11R/JAM-A, CD321), to examine its role in the early stages of metastasis.

F11R/JAM-A is the tight junction (TJ) protein important for platelet and leukocyte interactions with the epithelium and endothelium [5–12]. Upon inflammation, F11R/JAM-A is internalized from endothelial TJs into recycling endosomes and relocated to the apical endothelial surface that faces the vessel lumen [13]. F11R/JAM-A molecules are thereby able to interact with leukocyte surface proteins, including F11R/JAM-A (by *trans*-homodimerization) and integrins, thus promoting leukocyte TEM [6, 14–17]. Cancer cells cross the endothelial barrier in a similar manner to that of leukocytes [4].

The aberrant expression of F11R/JAM-A contributes to tumor progression [18] and its role is best studied in breast cancer [19–25]. However, the significance of F11R/JAM-A in breast cancer is controversial, since loss of F11R/JAM-A induces the breast cancer cell invasion [19–21], whereas the overexpression of this protein correlates with poor prognosis [22–25]. F11R/JAM-A was shown to promote TEM of monocytes in breast cancer [6] and to inhibit TEM of melanoma cells [15], but its direct effect on TEM of breast cancer cells has never been studied.

In order to define the role of F11R/JAM-A in breast cancer metastasis, we utilized a peptide derived from the sequence of the F11R/JAM-A protein, peptide 4D (P4D), which blocks the homophilic interactions between the F11R/JAM-A molecules. We investigated how P4D influences the interactions between the breast cancer cells and endothelial cells in the mouse breast cancer model, and examined its effects on human cell lines treated with thymosin β4 (Tβ4), an inducer of metastasis [26], and linked with breast cancer [27–29]. Further cell treatment involved the use of the inflammatory cytokines. Both the formation of TJs and the interactions of cancer cells with the endothelium were blocked by P4D. We propose that P4D can be considered as the drug candidate for use in the prevention of breast cancer metastasis.

## Methods

### Mice mammary gland tumor culture and tumor induction

Female BALB/c mice aged 8–12 weeks from the Animal House of the Institute of Biology, University of Bialystok (Bialystok, Poland) were housed under standard conditions at the Animal House of the Medical University of Lodz (Lodz, Poland). The procedures were conducted as per the National Animal Care Committee regulations, and were approved by the Local Ethical Committee for Animal Research in Lodz (license number 9/LB51/2017). All efforts were made to minimize animal suffering.

Murine 4T1 cells (American Type Culture Collection, ATCC) were cultured in RPMI-1640 with 10% fetal bovine serum (FBS) in flasks to 80% confluence. The 4T1 cells were harvested, resuspended at a concentration of 10^4^ cells in 100 µl PBS per mouse, and administered subcutaneously in inguinal nipple area. Seven days after the injections, the mice were divided into three groups: the Tβ4 group, the TGF-β1 group, and the control group (PBS). Non-control groups were given intraperitoneally Tβ4 or TGF-β1 resuspended in PBS at a concentration of 15 mg/1 kg body weight or 5 mg/1 kg body weight, respectively. Each injection was followed by 3 days of an injection-free period. After 21 days of treatment, the mice were anesthetized intraperitoneally with the ketamine and xylazine solution (0.1 ml/20 g mouse). Ten mice were used for each experimental group. Blood samples were collected by cardiac blood drawn using a syringe with K2-EDTA solution. Samples were centrifuged (662×*g*, 10 min, 4 °C), and the plasma was aliquoted and stored at − 80 °C for further analysis.

### Cell culture

MCF-10A (non-tumorigenic human breast epithelium), MCF-7 (human breast adenocarcinoma, Luminal A) [30], MDA-MB-231 (human breast adenocarcinoma, Claudin-low) [30], and HMEC-1 (human microvascular endothelial) cell lines were from ATCC. MCF-10A cells were grown in MEBM supplemented with MEGM Single Quots and cholera toxin (Lonza). MCF-7 and MDA-MB-231 cells were cultured in RPMI-1640 medium with 10% FBS. HMEC-1 cells were grown in MCDB 131 medium with 10 ng/mL epidermal growth factor (EGF), 1 µg/mL hydrocortisone, 10 mM glutamine, and 10% FBS. The cells were cultured in a humidified incubator (37 °C, 5% CO_2_). Confluent cells were passaged using trypsin–EDTA. The culture media were changed each 2–3 days.

### Cell treatment

The cells were cultured overnight in a serum-free medium and subsequently incubated for 0, 2, 5, 12, or 24 h with Tβ4 (100 nM), and then for 24 h with TGF-β1 (10 ng/ml), tumor necrosis factor-α (TNF-α; 10 ng/ml), or with a mixture of proinflammatory cytokines (TNF/IFN)—TNF-α (10 ng/ml) and interferon-γ (IFN-γ; 20 ng/ml). For the inhibition of TJ formation between the cancer cells and endothelium, the cells were treated with the P4D peptide with the sequence of a 70–82 amino acids fragment of the F11R/JAM-A mature polypeptide chain that is responsible for F11R/JAM-A *trans*-homodimerization [31]. The peptide P4D is a d-amino acid analog of the native peptide that binds to the 70–82 amino acid fragments and blocks the *trans*-homodimerization of F11R/JAM-A molecules, thus inhibiting the TJs formation in the endothelial monolayer as well as between the cancer cells and endothelial cells [32]. The sequence of control peptide (Scr) corresponded to P4D peptide, but was scrambled by random insertion of amino acid residues during the synthesis process. The peptides were synthesized and purified by LifeTein, LLC and their sequences were as follows: NH_2_-(dK)-SVT-(dR)-EDTGTYTC-CONH_2_ for P4D and NH_2_-S-(dK)-TVE-(dR)-TDTGTYC-OH for Scr. The cells were treated with P4D or Scr (500 µM) for 0, 1, 5, or 24 h. The cells incubated with forskolin (FSK; BioShop) for 24 h at a concentration of 10 µM served as the positive control of the junctions tightening for impedance-based measurements of endothelial permeability.

### Enzyme-linked immunosorbent assay

F11R/JAM-A levels in murine plasma were assayed by RayBio^®^ Mouse JAMA ELISA Kit (RayBiotech) according to the manufacturer’s protocol and measured spectrophotometrically using the Wallac 1420 VICTOR2 Multilabel Counter (PerkinElmer).

### RT-PCR

Total RNA was isolated from the cells with the TriPure Reagent (Roche) due to the manufacturer’s protocol, dissolved in nuclease-free water, and analyzed spectrophotometrically for concentration and purity. The RNA concentrations in all samples were equalized and RNA was transcribed to cDNA using the iScript cDNA Synthesis Kit (BioRad) according to the manufacturer’s instructions. The cDNA samples were amplified by the Dream Taq Green PCR Master Mix (Thermo) and the Whatman Biometra T3 Thermocycler (Biometra) according to the provided instructions. The specific oligonucleotide primer pairs were synthesized (Genomed, Warsaw, Poland) and used in PCR reactions for the following transcripts: forward 5′-CGAGAGGAAACTGTTGTGCC-3′ and reverse 5′-AACGAGTCTGGTGGTGTCTC-3′ for F11R/JAM-A, forward 5′-GAGAGATGATGACCCTTTTGGC-3′ and reverse 5′-CCATCACCATCTTCCCAGGAGCG-3′ for glyceraldehyde 3-phosphate dehydrogenase (GAPDH, used as the loading control). The amplified products were separated in 7% polyacrylamide gels in Tris–acetate-EDTA buffer (TAE; tris(hydroxymethyl)aminomethane; ethylenediaminetetraacetic acid). Bands were visualized by ethidium bromide and UV light and documented using Gel Doc 2000 (BioRad).

### Western blot

The cells were lysed with lysis buffer (1% Triton X-100, 0.1% SDS, and protease inhibitors in PBS). The protein concentrations were determined with the BCA Protein Assay Kit (Thermo). Cell lysates (50 µg proteins per sample) were boiled in the loading buffer (50 mM Tris–HCl, pH 6.8, 8% glycerol, 2% SDS, 5% β-mercaptoethanol, 0.002% bromophenol blue), separated under reducing conditions on 4–20% gradient SDS-PAGE gels (BioRad), and electroblotted (120 min, 200 mA, 4 °C) onto polyvinylidene difluoride (PVDF) membranes (BioRad). The membranes were blocked for 2 h at room temperature with Tris-buffered saline (TBS) containing 5% non-fat dry milk and incubated overnight at 4 °C with a primary antibody diluted in TBS containing 0.05% Tween 20 (TBST). The following primary antibodies were used: monoclonal rabbit anti-JAM-A (Abcam) and monoclonal mouse anti-GAPDH (R&D Systems). The membranes were washed with TBST and incubated for 1 h at room temperature with the corresponding HRP-conjugated secondary antibodies (Santa Cruz) and processed for chemiluminescent detection using Westar ηC ECL Substrate (Cyanagen). The images were documented by ChemiDoc MP Imager (BioRad) and analyzed densitometrically with ImageJ software (National Institute of Health, USA). GAPDH bands served as loading controls.

### Flow cytometry

The cells were labeled with the FITC anti-human CD321 (F11R/JAM-A) Mouse IgG_1_ Antibody or with the relevant isotype control (BioLegend). Labeled cells were washed, harvested with ice cold EDTA/PBS, fixed with BD CellFix (Becton–Dickinson), and analyzed by FACS Canto II flow cytometer (Becton–Dickinson) upon the fluorescence excitation at 488 nm and the emission at 517 nm. Data were recorded with DIVA (Becton–Dickinson) and analyzed using FCSalyzer software (https://sourceforge.net/projects/fcsalyzer/).

### Adhesion of breast cancer cells to endothelial monolayer

Two days after seeding, HMEC-1 cells have formed the confluent monolayers in the wells of 24-well plates and were subjected to a suitable treatment. Mammary epithelial cells (MECs) were starved overnight and on the assay day were labeled with CellTracker Green CMFDA Dye (Molecular Probes). Just before the assay, the HMEC-1 cells culture medium was substituted with the adhesion medium (MCDB 131 containing 1 mM CaCl_2_, 1 mM MgCl_2_, and 1% bovine serum albumin). Subsequently the aliquots of MECs (1 × 10^5^/well) were applied onto the endothelial monolayers and allowed to adhere for 1 h in a humidified incubator (37 °C, 5% CO_2_). Non-adherent cells were washed away by PBS. The adherent cells were lysed by 2% SDS, and the fluorescence was measured using the Wallac 1420 VICTOR2 Multilabel Counter at 492 nm of excitation and 517 nm of emission wavelengths.

### Transmigration of MECs across the endothelium

HMEC-1 cells were cultured in Transwell units with polycarbonate filter and 8 μm pores (Costar) in 24-well plates for 2 days at 37 °C to confluence. MECs were starved overnight and labeled with CellTracker Green CMFDA Dye. An aliquot (1 × 10^5^/well) of fluorescent MECs in serum-free medium was added in the top of the Transwell chamber, whereas the conditioned medium was used as a chemoattractant in the lower compartment. The wells containing the serum-free medium in the lower compartment were used to estimate the background migration. MECs were migrating across the endothelial monolayer for 6 h in a humidified incubator (temperature of 37 °C and 5% CO_2_ atmosphere). At the end of the assay, non-migrated cells were wiped out from the inner surface of the membrane with a cotton swab. The assay was documented at 200 × magnification with a fluorescent inverted microscope and a digital camera (Zeiss). The migrated cells (five randomly chosen fields of each well) were counted using the ImageJ software.

### Macromolecular permeability of endothelial monolayer

Endothelial permeability was analyzed by macromolecular tracer assay. HMEC-1 cells were grown in Transwell inserts with polyethylene terephthalate filters and 0.4-μm pores in 24-well plates at 1.5 × 10^5^ cells/insert and immediately treated with P4D or Scr peptide at a concentration of 500 μM (the samples indicated as 0 h of incubation). Non-treated cells served as a control sample (Ctrl). Moreover, HMEC-1 cells were treated with the peptides at 1, 5, or 24 h after seeding. One hour after the last treatment FITC-dextran with a molecular weight of 40 kDa was added into the inserts and the culture medium from the wells was replaced with PBS. After 1 h of incubation in darkness in a humidified incubator (37 °C, 5% CO_2_), the PBS from the wells (outside the inserts) was transferred into the wells of a black 96-well plate and the fluorescence was measured using the Wallac 1420 VICTOR2 Multilabel Counter at 492 nm of excitation and 517 nm of emission wavelengths.

### Impedance-based measurement of endothelial permeability

Experiments based on impedance measurements were performed at Bionanopark, Ltd. (Lodz, Poland) with the ACEA xCELLigence^®^ Real-Time Cell Analysis (RTCA) DP system (Roche) according to the manufacturer’s instructions. Briefly, HMEC-1 cells were seeded at 40,000 cells per well and the plates were locked into the RTCA instrument for continuous recording of impedance changes expressed as Cell Index (CI) values [33]. During the phase of logarithmic cell growth HMEC-1 cells were pretreated with cytokines (TNF/IFN) and 24 h later they were treated with the tested compounds (Scr, P4D, or FSK). Alternatively, the cells were treated with the compounds 2 h after the cytokine treatment or the compound treatment was applied about 3 h after HMEC-1 seeding and about 67 h later was followed by the cytokine treatment. For the sample designated as ‘P4D & TNF/IFN’ after the pretreatment with P4D, the cells were subjected to the concurrent treatment with P4D and cytokines.

### Statistical analysis

The results are presented as arithmetic mean ± standard deviation (SD) of at least three independent experiments. The data were analyzed by one-way ANOVA with the Tukey’s post hoc multiple comparisons test using the GraphPad Prism software (GraphPad Software). Differences were considered statistically relevant at *P* < 0.05.

## Results

### Tβ4 and TGF-β reduce the plasma level of soluble F11R/JAM-A

**Fig. 1:**
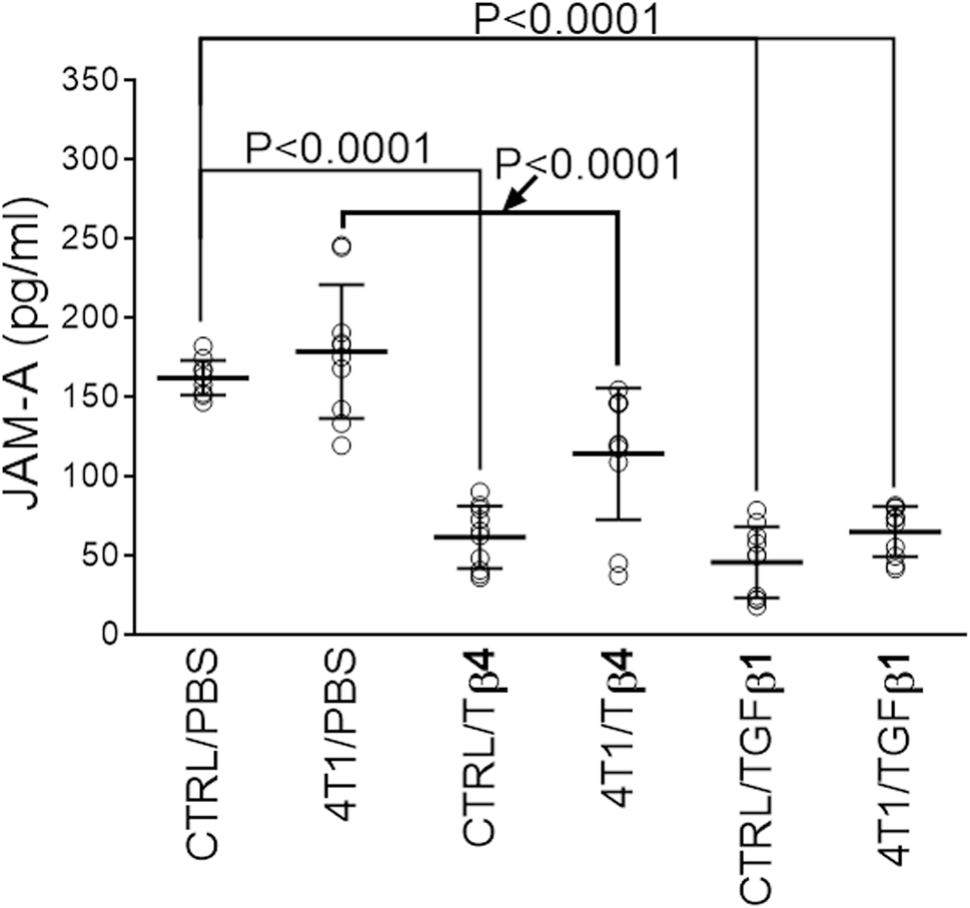
Levels of F11R/JAM-A antigen measured by ELISA in plasma from mice with (4T1) or without (CTRL) breast cancer induction and treated with Tβ4, TGF-β1, or vehicle (PBS) as described in Materials and methods Section. Results are expressed as arithmetic mean ± SD (*n* = 10). Statistical analysis was performed by one-way ANOVA with the Tukey’s multiple comparison post hoc test

Experiments were performed in vivo in the murine 4T1 breast cancer model [34–36]. Plasma samples obtained from the blood of 4T1-treated mice and untreated mice were analyzed by ELISA for soluble F11R/JAM-A (sJAM-A) quantitation. The injection of 4T1 cells did not change the level of sJAM-A in the murine plasma (Fig. 1). However, a significant decrease in the murine plasma level of sJAM-A was found upon the administration of either Tβ4 or TGF-β1 (Fig. 1 and Supplementary Table S1).

**Fig. 2:**
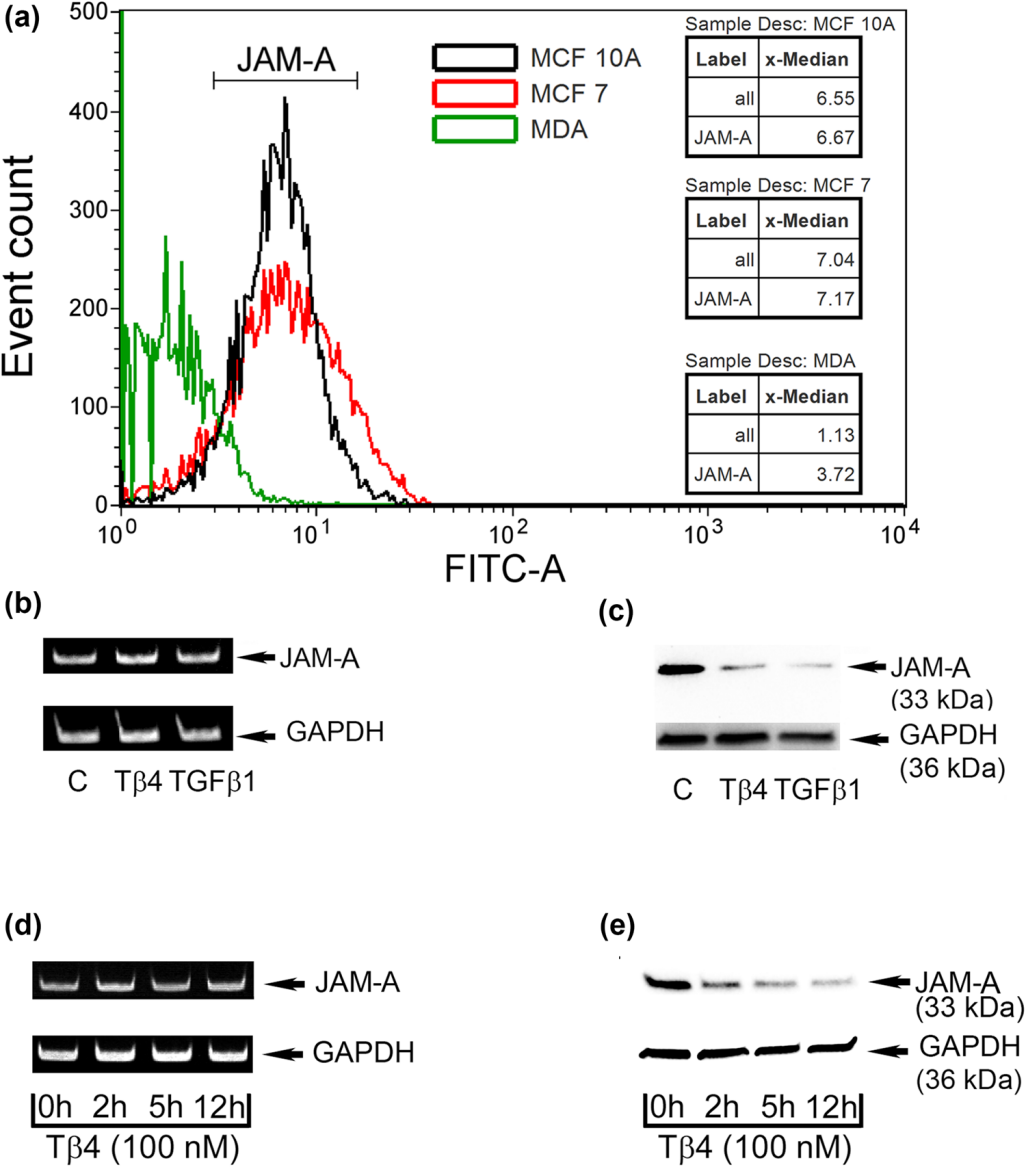
**a** F11R/JAM-A (CD321) cell surface expression in non-tumorigenic MECs MCF-10A (black color) or in breast cancer cell lines (MCF-7, red color, and MDA-MB-231, green color) detected by flow cytometry. RT-PCR (**b**) and Western blot (**c**) assay for the detection of F11R/JAM-A expression levels using the RNA and protein extracts from HMEC-1 cells non-treated (C; control cells) or treated for 24 h with Tβ4 at a concentration of 200 nM or with TGF-β1 at a concentration of 10 ng/ml. RT-PCR (**d**) and Western blot (**e**) assay for the detection of F11R/JAM-A expression levels using the RNA and protein extracts from HMEC-1 cells incubated with Tβ4 at a concentration of 200 nM for 0, 2, 5, or 12 h. Figure presents the representative results of 3 independent experiments

The expression of F11R/JAM-A was examined on non-tumorigenic human breast epithelial cells and on tumorigenic cells by flow cytometry (Fig. 2a). The lowest level of F11R/JAM-A/CD321 was detected on the surface of tumorigenic MDA-MB-231 cells (green). The tumorigenic MCF-7 cells and the non-tumorigenic MCF-10A cells exhibited similar F11R/JAM-A protein levels (Fig. 2a).

The administration of Tβ4 or TGF-β1 significantly dropped the F11R/JAM-A levels in the murine plasma (Fig. 1). Consequently, we examined whether these molecules could affect the expression of F11R/JAM-A on the protein and/or message level. The presence of either Tβ4 or TGF-β1 did not alter F11R/JAM-A message in treated cells (Fig. 2b, d). However, Tβ4 significantly decreased the level of the F11R/JAM-A protein in HMEC-1 cells after a 24-hour incubation period similar to TGF-β1 that was used as the positive control for demonstrating the F11R/JAM-A downregulation (Fig. 2c) [20]. The incubation of cells with Tβ4 for up to a 12-hr period of time resulted in significant time-dependent changes in the F11R/JAM-A protein level (Fig. 2e).

**Fig. 3:**
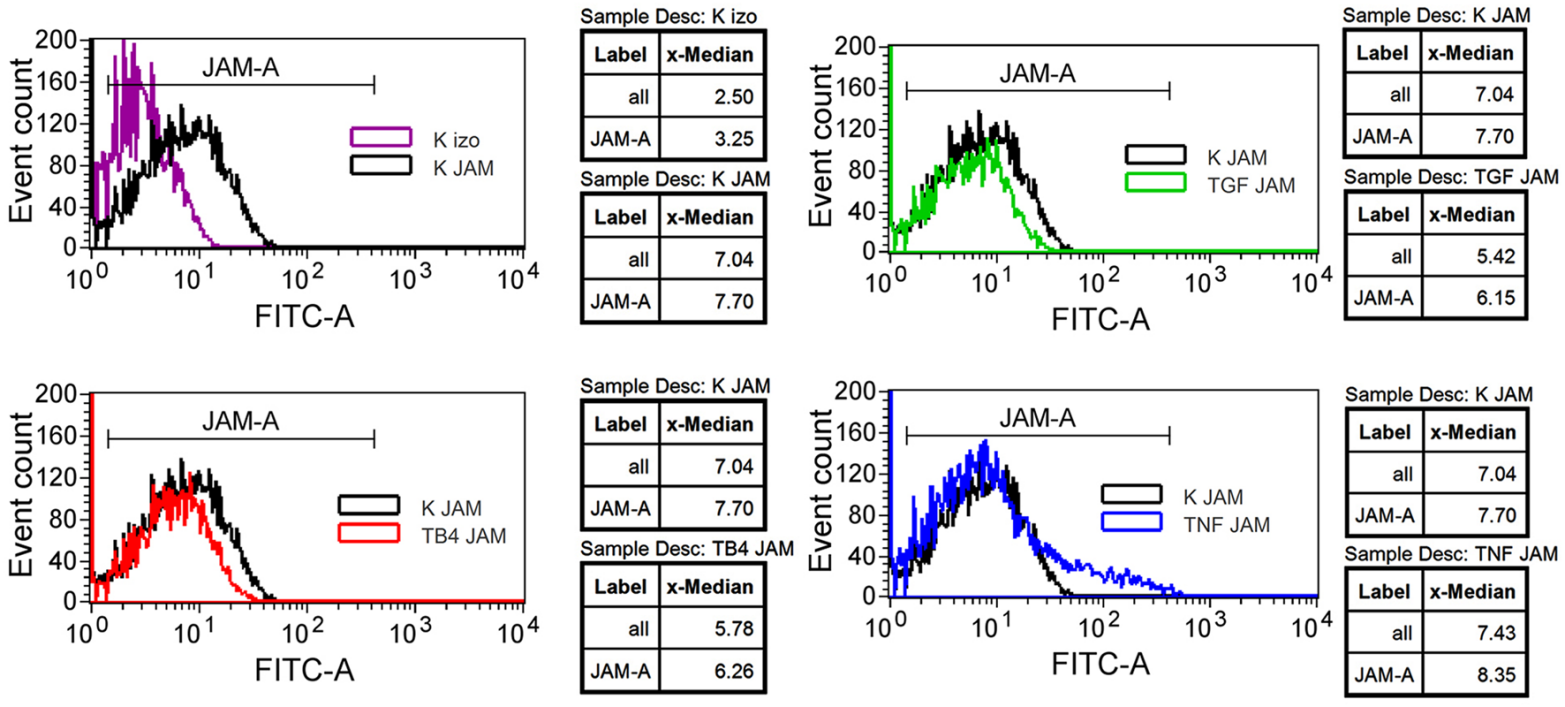
Flow cytometry analysis of F11R/JAM-A (CD321) expression on the surface of HMEC-1 cells. The cells were untreated (Control, K, black color) or treated with 200 nM of thymosin β4 (TB4, red color), 10 ng/ml of TGF-β1 (TGF, green color), or 10 ng/ml of TNF-α (TNF, blue color) for 24 h. For the superficial visualization of F11R/JAM-A antigen, the cells were labeled with FITC anti-human CD321 Mouse IgG_1_ Antibody (JAM) or with the corresponding isotype control (izo, purple color). The representative results of three independent experiments are shown

Any changes in the F11R/JAM-A/CD321 protein expression on HMEC-1 cells treated with Tβ4, TGF-β1, or TNF-α were analyzed by flow cytometry. The presence of Tβ4 (red) and TGF-β1 (green) decreased the amount of the F11R/JAM-A protein (Fig. 3). However, TNF-α slightly increased the F11R/JAM-A/CD321 protein level (blue).

### F11R/JAM-A derived peptide 4D (P4D) blocks TEM of breast cancer cells

**Fig. 4:**
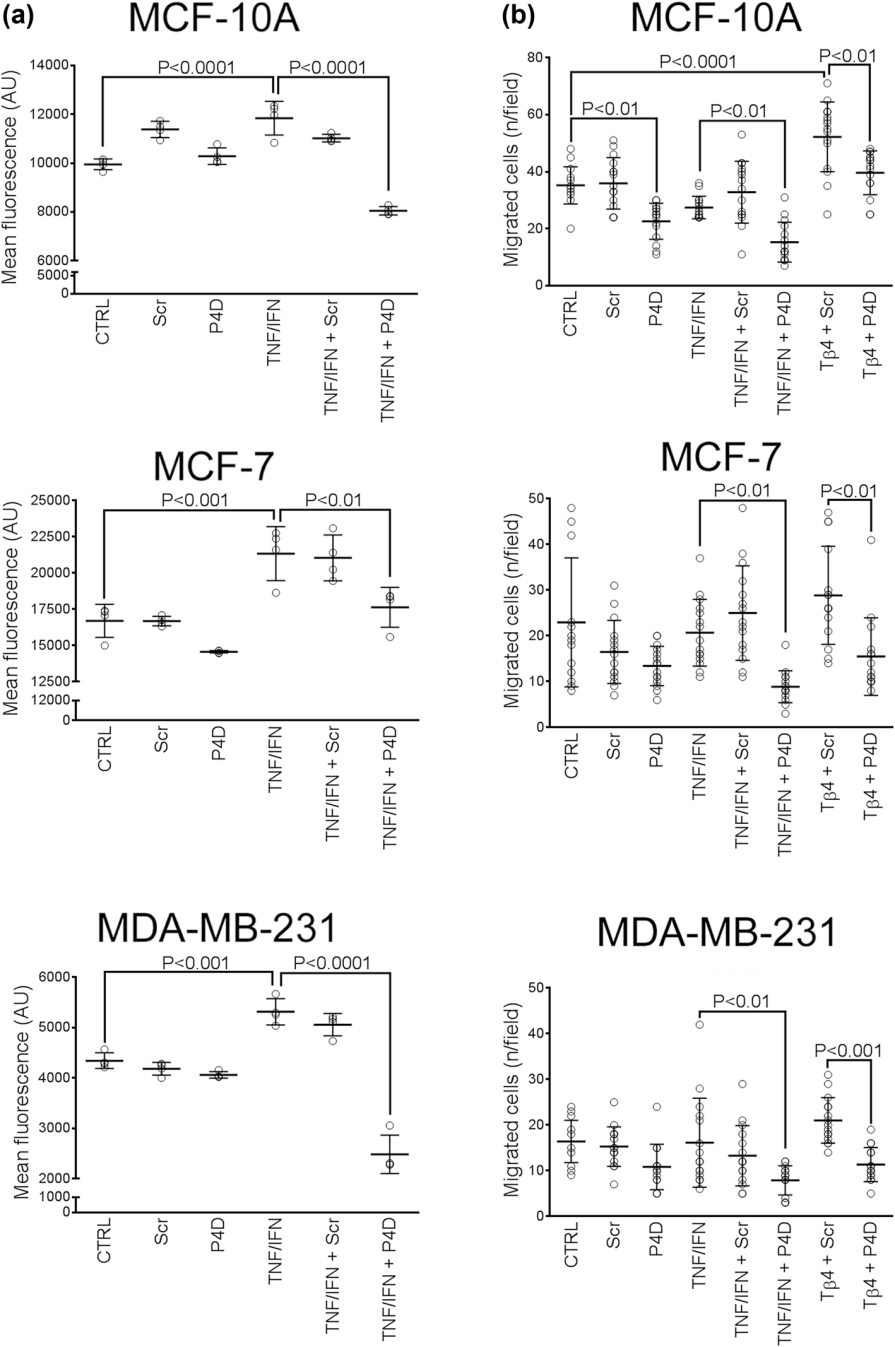
Effect of F11R/JAM-A derived peptide P4D on breast cancer cell interactions with endothelium. **a** Adhesion of breast cancer cells to HMEC-1 cells. Results are expressed as mean fluorescence of adhered cells ± SD (*n* = 4). **b** TEM of breast cancer cells. The plots present the mean values of migrated cell number per field of view ± SD (*n* = 15). *CTRL* control sample (untreated cells), *Scr* scrambled P4D peptide, *P4D* F11R/JAM-A antagonistic peptide (500 μM, 4-h incubation), *TNF/IFN* preincubation with TNF-α (10 ng/ml) and IFN-γ (20 ng/ml) for 24 h before the treatment with P4D peptides, *Tβ4* preincubation with Tβ4 (200 nM) for 24 h before the treatment with P4D peptides. Cell migration towards serum-free medium was subtracted from the corresponding numbers of cells migrated towards conditioned medium

The soluble form of F11R/JAM-A was reported to inhibit both the human platelet aggregation induced by a stimulatory monoclonal antibody M.Ab.F11 and the platelet adhesion to the inflamed endothelium [5, 7, 10, 31]. The F11R/JAM-A antagonistic peptide 4D (P4D) also blocked the M.Ab.F11-induced aggregation and the adhesion of platelets to activated endothelial cells. Consequently, we speculated whether the decrease in the plasma level of sJAM-A produced by Tβ4/TGF-β1 could interfere with adhesive processes and thereby promote the transendothelial migration (TEM) of breast cancer cells. Thus, we examined the interactions between mammary epithelial cells (MECs) and endothelial cells in the presence of the P4D peptide (Fig. 4).

The cytokines TNF-α and IFN-γ (TNF/IFN) enhanced the adhesion of MECs to the endothelium (Fig. 4a). The addition of P4D significantly inhibited the adhesion of MECs to the inflamed endothelium and this effect was the most pronounced for MDA-MB-231 cells (Fig. 4a). Likewise, we observed that the TEM of breast cancer cells in the presence of TNF/IFN and Tβ4 was blocked by P4D (Fig. 4b and Supplementary Fig. S1). Tβ4 activates the cellular motility and inhibits the adhesion [37], thus we did not utilize this peptide for adhesion assays.

### Peptide 4D displays the barrier-protecting effect on endothelial monolayer

**Fig. 5:**
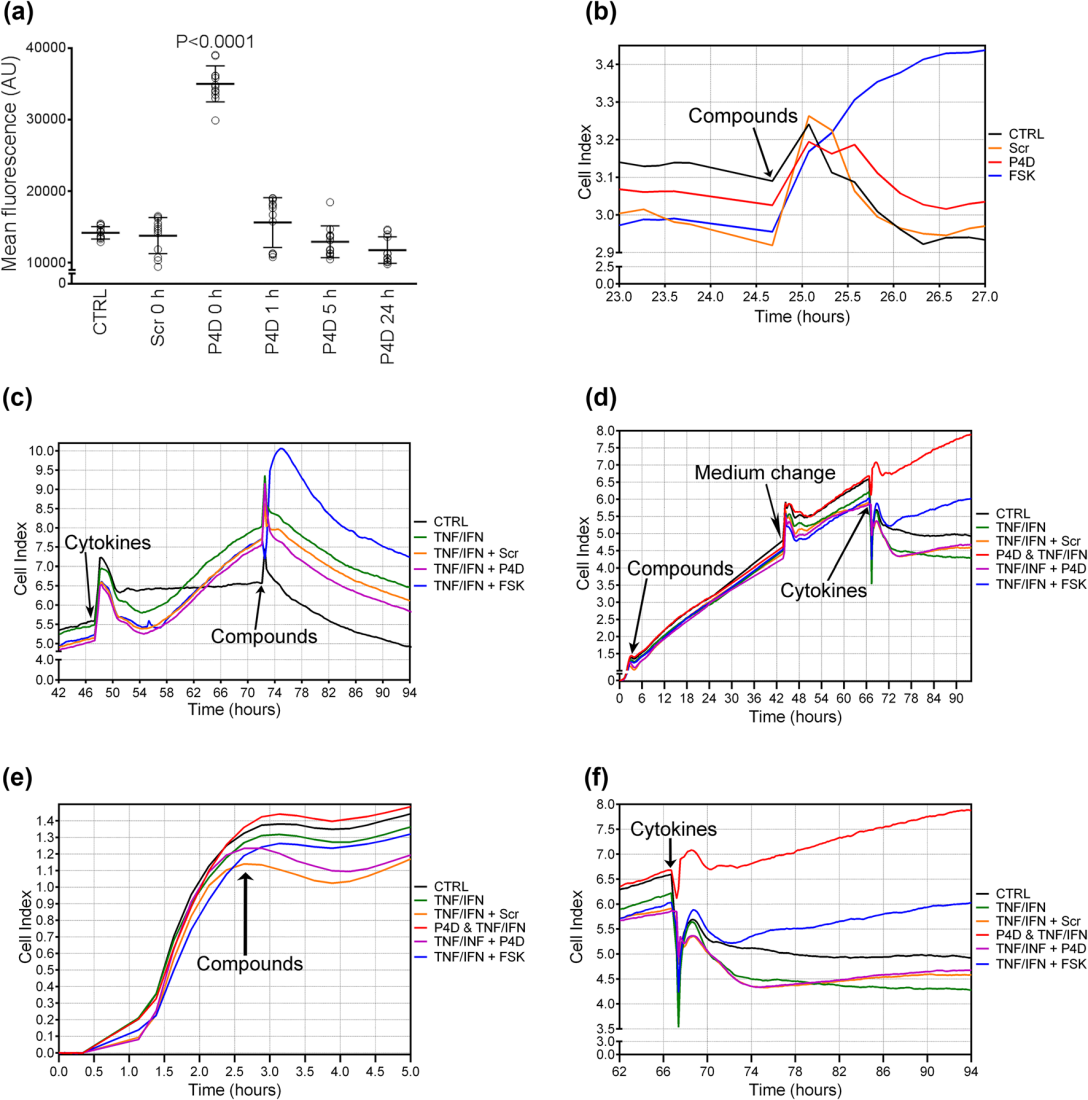
Permeability of HMEC-1 endothelial monolayer. **a** Transendothelial flux of 40-kda FITC-dextran across HMEC-1 monolayer was measured with a Transwell^®^ two-compartment system. HMEC-1 cells were treated with P4D peptide or with Scrambled P4D peptide (Scr) at a concentration of 500 μM at specified periods of time after seeding. Non-treated cells served as a control sample (CTRL). The plot presents the arithmetic mean ± SD (*n* = 12). Graphs on panels (**b**–**f)** present the results of impedance-based real time measurements of HMEC-1 monolayer permeability shown as the time courses of cytokine-induced and compound-induced alterations of CI levels. The cells were treated with the tested cytokines or compounds at the time points marked with arrows. Control cells (CTRL) were left non-treated and the alterations of their CI values are presented with the black line. **b** HMEC-1 cells were treated with the tested compounds (P4D, Scr, or FSK) ca. 24 h after plating. **c** HMEC-1 monolayer was subjected to cytokine (TNF/IFN) treatment ca 48 h after seeding and subsequently to compounds treatment ca. 72 h after seeding. **d**–**f** About 2.5 h after seeding HMEC-1 cells were subjected to the compounds treatment followed by the cytokine treatment ca. 67 h after plating. **e** presents the detailed view of CI levels alterations after the compound treatment, whereas (**f**) emphasizes the effect of cytokines on the changes in CI values after the compound pretreatment for > 60 h. P4D & TNF/IFN: ca. 2.5 h after seeding, the cells were treated with P4D and next ca. 67 h after plating, the cells were subjected to the concurrent treatment with P4D and cytokines

Subsequently, we examined whether P4D affects the endothelial barrier permeability (Fig. 5). Transendothelial flux of 40-kDa FITC-dextran was elevated only when P4D was applied immediately (at 0 h) after the HMEC-1 cells were seeded (Fig. 5a). We conclude that P4D prevented de novo formation of TJs without disrupting the pre-existing ones.

The effect of P4D on endothelial barrier was monitored by the Real-Time Cell Analysis (RTCA). The P4D did not affect the endothelial permeability when applied ~ 24 h after the cell seeding or 24 h after the cytokine treatment (Fig. 5b, c). Therefore, we treated the HMEC-1 monolayer with P4D, Scr, or FSK 2.5 h after plating when the cells adhered to the bottom of the wells without forming a confluent monolayer, as demonstrated by the low CI levels (Fig. 5d, e). Presumably, at this time period, the TJs have not fully developed and only a minor fraction of F11R/JAM-A molecules have formed *trans*-homophilic interactions. The endothelium was treated with inflammatory cytokines ~ 60 h later, during the logarithmic growth phase (Fig. 5d, f). The cells from ‘P4D & TNF/IFN’ sample were retreated with P4D concurrently with TNF/IFN treatment. The barrier-protecting effect of P4D was more evident than that of FSK, when P4D pretreatment was followed by the second supplementary dose applied simultaneously with the cytokines (Fig. 5d, f). Otherwise, P4D did not reveal any barrier-protecting properties without the supplementary dose (Fig. 5f).

## Discussion

Our studies on the murine 4T1 breast cancer model demonstrated that administration of Tβ4 and TGF-β1 decreased the sJAM-A levels in murine blood. As the F11R mRNA level in HMEC-1 cells did not change, these observations suggest the reduced “shedding” of the F11R/JAM-A protein from the endothelial cell surface to the blood [38], but not downregulated protein expression [20]. F11R/JAM-A shedding occurs predominantly upon inflammation, when sJAM-A limits the leukocyte recruitment to inflammation sites [38]. The reduced shedding of F11R/JAM-A and decrease in protein expression could result from TGF-β1-induced lysosomal degradation of F11R/JAM-A [39]. Tβ4 probably induced the lysosomal proteolysis of F11R/JAM-A in a similar mode to TGF-β1.

The lowered F11R/JAM-A expression in MDA-MB-231 cells as compared with two other MEC lines (Fig. 4a) is in accordance with the previously reported negative correlation of JAM-A expression with the metastatic potential of breast cancer cells [19, 20].

Decreased plasma level of sJAM-A was suspected to promote the adhesive interactions of breast cancer cells with endothelium. Adhesion is a substantial early step of TEM [4, 14]. Moreover, inflammatory cytokines increase the leukocyte adhesion to endothelium, thus triggering F11R-dependent TEM [40]. Consequently, we employed the F11R/JAM-A antagonistic peptide 4D [31, 32] that blocked the breast cancer cell adhesion to the inflamed endothelium, which was particularly evident for MDA-MB-231 cells (Fig. 4a). We (Fig. 2a) and other authors [19, 20] have shown that this cell line expresses low JAM-A levels as compared with other MECs. Thus MDA-MB-231 cells could be more sensitive to the function of P4D. Migratory ability of MDA-MB-231 cells may be increased as compared with MCF-7 cells due to the lowered level of F11R/JAM-A [19–21]. The strongest P4D-dependent reduction of cytokine-stimulated TEM was observed for high JAM-A expressing MCF-7 cells (Fig. 4b). This negates the inverse relation between the JAM-A expression and breast cancer cell motility [19–21] and confirms that JAM-A drives the breast cancer cell migration [22–25]. Moreover, P4D inhibited the Tβ4-induced TEM of breast cancer cells (Fig. 4b). The upregulated TEM of MCF-10A cells confirms that these cells may not represent a perfect model for non-tumorigenic mammary epithelium [41].

P4D abrogated the TJs formation without the endothelial monolayer breakdown (Fig. 5a). The barrier-protecting effect of P4D was enhanced when P4D was applied shortly after the cytokine treatment and P4D administration was repeated with a booster dose (Fig. 5b–f). Concurrently, the inflammatory cytokines enhanced the endothelial permeability (Fig. 5c). Accordingly, TNF-α increased the endothelial permeability, while F11R/JAM-A was removed from TJs and/or internalized [42]. Furthermore, TNF-α with TGF-β3 significantly accelerated the JAM-A internalization [43].

F11R/JAM-A derived peptides were previously applied for the drug development in the cardiovascular disorders treatment [7, 10, 31, 32, 44]. Here, we report that Tβ4 and TGF-β1 decrease the plasma levels of sJAM-A in the murine 4T1 model. Soluble F11R/JAM-A inhibits M.Ab.F11-induced platelet aggregation and adhesion to the inflamed endothelium [7, 10, 31] and attenuates the TEM of neutrophils [38]. Thus P4D is able to restore the sJAM-A function. Accordingly, P4D abrogates de novo formation of TJ as demonstrated by the inhibition of breast cancer cell adhesion to the inflamed endothelium and by the suppression of breast cancer cell TEM.

We demonstrate the role of F11R/JAM-A in breast cancer metastasis. Tumor inducers Tβ4 and TGF-β1 decrease the plasma levels of soluble F11R/JAM-A. This upregulates the interactions between the cancer cells and the endothelium leading to metastasis. The decreased level of sJAM-A can be restored by P4D which blocks adhesion and TEM of breast cancer cells. Thus we suggest that P4D can be considered as a blocker of breast cancer cell extravasation. Nevertheless, in vivo and clinical studies are needed to test the effectiveness of P4D as an anti-metastatic drug.

## Electronic supplementary material

Below is the link to the electronic supplementary material.

Supplementary material 1 (DOC 216 kb)

## Data Availability

Data supporting these results are available from the corresponding author upon request.

## Abbreviations

BCA: Bicinchoninic acid
CTRL: Control
ELISA: Enzyme-linked immunosorbent assay
FBS: Fetal bovine serum
FITC: Fluorescein isothiocyanate
FSK: Forskolin
GAPDH: Glyceraldehyde 3-phosphate dehydrogenase
HRP: Horseradish peroxidase
IFN-γ: Interferon-γ
JAM-A: Junctional adhesion molecule-A
K2-EDTA: Dipotassium ethylenediaminetetraacetic acid
M.Ab.F11: A potent stimulatory monoclonal antibody that induces platelet secretion followed by aggregation
MEC: Mammary epithelial cell
P4D: F11R/JAM-A derived peptide 4D
PBS: Phosphate-buffered saline
PVDF: Polyvinylidene difluoride
qRT-PCR: Quantitative reverse transcription-polymerase chain reaction
RTCA: Real-Time Cell Analysis
Scr: F11R/JAM-A derived scrambled peptide 4D
SDS: Sodium dodecyl sulphate
sJAM-A: Soluble JAM-A
TBS: Tris-buffered saline
Tβ4: Thymosin β4
TEM: Transendothelial migration
TGF-β1: Transforming growth factor-β1
TJ: Tight junction
TNF-α: Tumor necrosis factor-α

## References

[1] Torre LA, Bray F, Siegel RL, Ferlay J, Lortet-Tieulent J, Jemal A (2015). Global cancer statistics, 2012. CA Cancer J Clin.

[2] Wojciechowska U, Didkowska J. Zachorowania i zgony na nowotwory złośliwe w Polsce. Nowotwory piersi u kobiet (C50). In: Krajowy Rejestr Nowotworów, Centrum Onkologii-Instytut im. Marii Skłodowskiej-Curie. http://onkologia.org.pl/nowotwory-piersi-kobiet/. Accessed 28 April 2018

[3] Allen MD, Jones LJ (2015). The role of inflammation in progression of breast cancer: friend or foe?. Int J Oncol.

[4] Reymond N, d’Água BB, Ridley AJ (2013). Crossing the endothelial barrier during metastasis. Nat Rev Cancer.

[5] Kornecki E, Walkowiak B, Naik UP, Ehrlich YH (1990). Activation of human platelets by a stimulatory monoclonal antibody. J Biol Chem.

[6] Martin-Padura I, Lostaglio S, Schneemann M, Williams L, Romano M, Fruscella P, Panzeri C, Stoppacciaro A, Ruco L, Villa A, Simmons D, Dejana E (1998). Junctional adhesion molecule, a novel member of the immunoglobulin superfamily that distributes at intercellular junctions and modulates monocyte transmigration. J Cell Biol.

[7] Babinska A, Kedees MH, Athar H, Ahmed T, Batuman O, Ehrlich YH, Hussain MM, Kornecki E (2002). F11-receptor (F11R/JAM) mediates platelet adhesion to endothelial cells: role in inflammatory thrombosis. Thromb Haemost.

[8] Ong KL, Leung RY, Babinska A, Salifu MO, Ehrlich YH, Kornecki E, Wong LY, Tso AW, Cherny SS, Sham PC, Lam TH, Lam KS, Cheung BM (2009). Elevated plasma level of soluble F11 receptor/junctional adhesion molecule-A (F11R/JAM-A) in hypertension. Am J Hypertens.

[9] Sladojevic N, Stamatovic SM, Keep RF, Grailer JJ, Sarma JV, Ward PA, Andjelkovic AV (2014). Inhibition of junctional adhesion molecule-A/LFA interaction attenuates leukocyte trafficking and inflammation in brain ischemia/reperfusion injury. Neurobiol Dis.

[10] Babinska A, Azari BM, Salifu MO, Liu R, Jiang XC, Sobocka MB, Boo D, Al Khoury G, Deitch JS, Marmur JD, Ehrlich YH, Kornecki E (2007). The F11 receptor (F11R/JAM-A) in atherothrombosis: overexpression of F11R in atherosclerotic plaques. Thromb Haemost.

[11] Cavusoglu E, Kornecki E, Sobocka MB, Babinska A, Ehrlich YH, Chopra V, Yanamadala S, Ruwende C, Salifu MO, Clark LT, Eng C, Pinsky DJ, Marmur JD (2007). Association of plasma levels of F11 receptor/junctional adhesion molecule-A (F11R/JAM-A) with human atherosclerosis. J Am Coll Cardiol.

[12] Azari BM, Marmur JD, Salifu MO, Ehrlich YH, Kornecki E, Babinska A (2011). Transcription and translation of human F11R gene are required for an initial step of atherogenesis induced by inflammatory cytokines. J Transl Med.

[13] Stamatovic SM, Sladojevic N, Keep RF, Andjelkovic AV (2012). Relocalization of junctional adhesion molecule A during inflammatory stimulation of brain endothelial cells. Mol Cell Biol.

[14] Garrido-Urbani S, Bradfield PF, Imhof BA (2014). Tight junction dynamics: the role of junctional adhesion molecules (JAMs). Cell Tissue Res.

[15] Ghislin S, Obino D, Middendorp S, Boggetto N, Alcaide-Loridan C, Deshayes F (2011). Junctional adhesion molecules are required for melanoma cell lines transendothelial migration in vitro. Pigment Cell Melanoma Res.

[16] Offiah G, Brennan K, Hopkins AM, Gunduz M, Gunduz E (2011). Junctional adhesion molecules (JAMs)-new players in breast cancer?. Breast cancer-focusing tumor microenvironment, stem cells and metastasis.

[17] Ostermann G, Weber KS, Zernecke A, Schröder A, Weber C (2002). JAM-1 is a ligand of the β_2_ integrin LFA-1 involved in transendothelial migration of leukocytes. Nat Immunol.

[18] Zhao C, Lu F, Chen H, Zhao X, Sun J, Chen H (2014). Dysregulation of JAM-A plays an important role in human tumor progression. Int J Clin Exp Pathol.

[19] Naik MU, Naik TU, Suckow AT, Duncan MK, Naik UP (2008). Attenuation of junctional adhesion molecule-A is a contributing factor for breast cancer cell invasion. Cancer Res.

[20] Wang Y, Lui WY (2012). Transforming growth factor-β1 attenuates junctional adhesion molecule-A and contributes to breast cancer cell invasion. Eur J Cancer.

[21] Cao M, Nie W, Li J, Zhang Y, Yan X, Guan X, Chen X, Zen K, Zhang CY, Jiang X, Hou D (2014). MicroRNA-495 induces breast cancer cell migration by targeting JAM-A. Protein Cell.

[22] McSherry EA, McGee SF, Jirstrom K, Doyle EM, Brennan DJ, Landberg G, Dervan PA, Hopkins AM, Gallagher WM (2009). JAM-A expression positively correlates with poor prognosis in breast cancer patients. Int J Cancer.

[23] McSherry EA, Brennan K, Hudson L, Hill AD, Hopkins AM (2011). Breast cancer cell migration is regulated through junctional adhesion molecule-A-mediated activation of Rap1 GTPase. Breast Cancer Res.

[24] Gotte M, Mohr C, Koo CY, Stock C, Vaske AK, Viola M, Ibrahim SA, Peddibhotla S, Teng YH, Low JY, Ebnet K, Kiesel L, Yip GW (2010). miR-145-dependent targeting of junctional adhesion molecule A and modulation of fascin expression are associated with reduced breast cancer cell motility and invasiveness. Oncogene.

[25] Murakami M, Giampietro C, Giannotta M, Corada M, Torselli I, Orsenigo F, Cocito A, d’Ario G, Mazzarol G, Confalonieri S, Di Fiore PP, Dejana E (2011). Abrogation of junctional adhesion molecule-A expression induces cell apoptosis and reduces breast cancer progression. PLoS ONE.

[26] Sribenja S, Wongkham S, Wongkham C, Yao Q, Chen C (2013). Roles and mechanisms of β-thymosins in cell migration and cancer metastasis: an update. Cancer Invest.

[27] Larsson LI, Holck S (2007). Occurrence of thymosin beta4 in human breast cancer cells and in other cell types of the tumor microenvironment. Hum Pathol.

[28] Yoon SY, Lee HR, Park Y, Kim JH, Kim SY, Yoon SR, Lee WJ, Cho BJ, Min H, Bang JW, Park H, Bang SI, Cho D (2011). Thymosin β4 expression correlates with lymph node metastasis through hypoxia inducible factor-α induction in breast cancer. Oncol Rep.

[29] Larsson LI, Holck S (2007). Localization of thymosin β-4 in tumors. Ann NY Acad Sci.

[30] Holliday DL, Speirs V (2011). Choosing the right cell line for breast cancer research. Breast Cancer Res.

[31] Babinska A, Kedees MH, Athar H, Sobocki T, Sobocka MB, Ahmed T, Ehrlich YH, Hussain MM, Kornecki E (2002). Two regions of the human platelet F11-receptor (F11R) are critical for platelet aggregation, potentiation and adhesion. Thromb Haemost.

[32] Babinska A, Clement CC, Swiatkowska M, Szymanski J, Shon A, Ehrlich YH, Kornecki E, Salifu MO (2014). Development of new antiatherosclerotic and antithrombotic drugs utilizing F11 receptor (F11R/JAM-A) peptides. Biopolymers.

[33] Sun M, Fu H, Cheng H, Cao Q, Zhao Y, Mou X, Zhang X, Liu X, Ke Y (2012). A dynamic real-time method for monitoring epithelial barrier function in vitro. Anal Biochem.

[34] Tao K, Fang M, Alroy J, Sahagian GG (2008). Imagable 4T1 model for the study of late stage breast cancer. BMC Cancer.

[35] Dalaklioglu S, Tasatargil A, Kale S, Tanriover G, Dilmac S, Erin N (2013). Metastatic breast carcinoma induces vascular endothelial dysfunction in Balb-c mice: role of the tumor necrosis factor-α and NADPH oxidase. Vascul Pharmacol.

[36] Pacia MZ, Buczek E, Blazejczyk A, Gregorius A, Wietrzyk J, Chlopicki S, Baranska M, Kaczor A (2016). 3D Raman imaging of systemic endothelial dysfunction in the murine model of metastatic breast cancer. Anal Bioanal Chem.

[37] Kozaczuk A, Selmi A, Bednarek R (2013). Bacterial expression, purification and angiogenesis-promoting activity of human thymosin β4. Protein Expr Purif.

[38] Koenen RR, Pruessmeyer J, Soehnlein O, Fraemohs L, Zernecke A, Schwarz N, Reiss K, Sarabi A, Lindbom L, Hackeng TM, Weber C, Ludwig A (2009). Regulated release and functional modulation of junctional adhesion molecule A by disintegrin metalloproteinases. Blood.

[39] Kern U, Wischnewski V, Biniossek ML, Schilling O, Reinheckel T (2015). Lysosomal protein turnover contributes to the acquisition of TGFβ-1 induced invasive properties of mammary cancer cells. Mol Cancer.

[40] Jaczewska J, Abdulreda MH, Yau CY, Schmitt MM, Schubert I, Berggren PO, Weber C, Koenen RR, Moy VT, Wojcikiewicz EP (2014). TNF-α and IFN-γ promote lymphocyte adhesion to endothelial junctional regions facilitating transendothelial migration. J Leukoc Biol.

[41] Qu Y, Han B, Yu Y, Yao W, Bose S, Karlan BY, Giuliano AE, Cui X (2015). Evaluation of MCF10A as a reliable model for normal human mammary epithelial cells. PLoS ONE.

[42] McKenzie JA, Ridley AJ (2007). Roles of Rho/ROCK and MLCK in TNF-alpha-induced changes in endothelial morphology and permeability. J Cell Physiol.

[43] Xia W, Wong EW, Mruk DD, Cheng CY (2009). TGF-beta3 and TNFalpha perturb blood-testis barrier (BTB) dynamics by accelerating the clathrin-mediated endocytosis of integral membrane proteins: a new concept of BTB regulation during spermatogenesis. Dev Biol.

[44] Babinska A, Clement CC, Przygodzki T, Talar M, Li Y, Braun M, Wzorek J, Swiatkowska M, Ehrlich YH, Kornecki E, Watala C, Salifu MO (2019). A peptide antagonist of F11R/JAM-A reduces plaque formation and prolongs survival in an animal model of atherosclerosis. Atherosclerosis.

