# Supplementary material for: Functional inhibition of F11 receptor (F11R/junctional adhesion molecule-A/JAM-A) activity by a F11R-derived peptide in breast cancer and its microenvironment
Source: Breast Cancer Res Treat. 2019 Oct 24;179(2):325–35. doi: 10.1007/s10549-019-05471-x (PMC6987052; doi:10.1007/s10549-019-05471-x)

**Supplemental materials**

**Functional inhibition of F11 Receptor (F11R/Junctional Adhesion Molecule-A/JAM-A) activity by a F11R-derived peptide in breast cancer and its microenvironment**

Radoslaw Bednarek ^1*^, Anna Selmi ^1^, Dagmara Wojkowska ^2^, Kamil Karolczak ^2^, Marcin Popielarski ^1^, Marta Stasiak ^1^, Moro O.Salifu ^3^, Anna Babinska ^3^, Maria Swiatkowska ^1^

^1^Department of Cytobiology and Proteomics, Medical University of Lodz, Lodz, Poland

^2^Department of Haemostasis and Haemostatic Disorders, Medical University of Lodz, Lodz, Poland

^3^Department of Medicine, State University of New York, Downstate Medical Center, Brooklyn, New York, USA

*correspondence: radoslaw.bednarek@umed.lodz.pl

Table S1. Statistical analysis of F11R/JAM-A antigen levels measured by ELISA in murine plasma (one-way ANOVA followed by Tukey’s multiple comparisons test). Statistically significant differences are marked with asterisks (** for P < 0.01, and **** for P < 0.0001).

| Tukey's multiple comparisons test | Probability value |
| --- | --- |
| CTRL/PBS vs. 4T1/PBS | 0.7766 |
| CTRL/PBS vs. CTRL/Tβ4 | < 0.0001 **** |
| CTRL/PBS vs. 4T1/Tβ4 | 0.0048 ** |
| CTRL/PBS vs. CTRL/TGFβ1 | < 0.0001 **** |
| CTRL/PBS vs. 4T1/TGFβ1 | < 0.0001 **** |
| 4T1/PBS vs. CTRL/Tβ4 | < 0.0001 **** |
| 4T1/PBS vs. 4T1/Tβ4 | < 0.0001 **** |
| 4T1/PBS vs. CTRL/TGFβ1 | < 0.0001 **** |
| 4T1/PBS vs. 4T1/TGFβ1 | < 0.0001 **** |
| CTRL/Tβ4 vs. 4T1/Tβ4 | 0.0015 ** |
| CTRL/Tβ4 vs. CTRL/TGFβ1 | 0.8055 |
| CTRL/Tβ4 vs. 4T1/TGFβ1 | 0.9998 |
| 4T1/Tβ4 vs. CTRL/TGFβ1 | < 0.0001 **** |
| 4T1/Tβ4 vs. 4T1/TGFβ1 | 0.0036 ** |
| CTRL/TGFβ1 vs. 4T1/TGFβ1 | 0.6440 |

Figure S1. TEM of breast cancer cells. Microscopic photographs presenting the fluorescently labeled breast cancer cells (MCF-7, MDA-MB-231) or non-tumorigenic MECs (MCF-10A) those transmigrated across the endothelial monolayer. The corresponding plots and detailed specifications are shown in Fig. 4b.


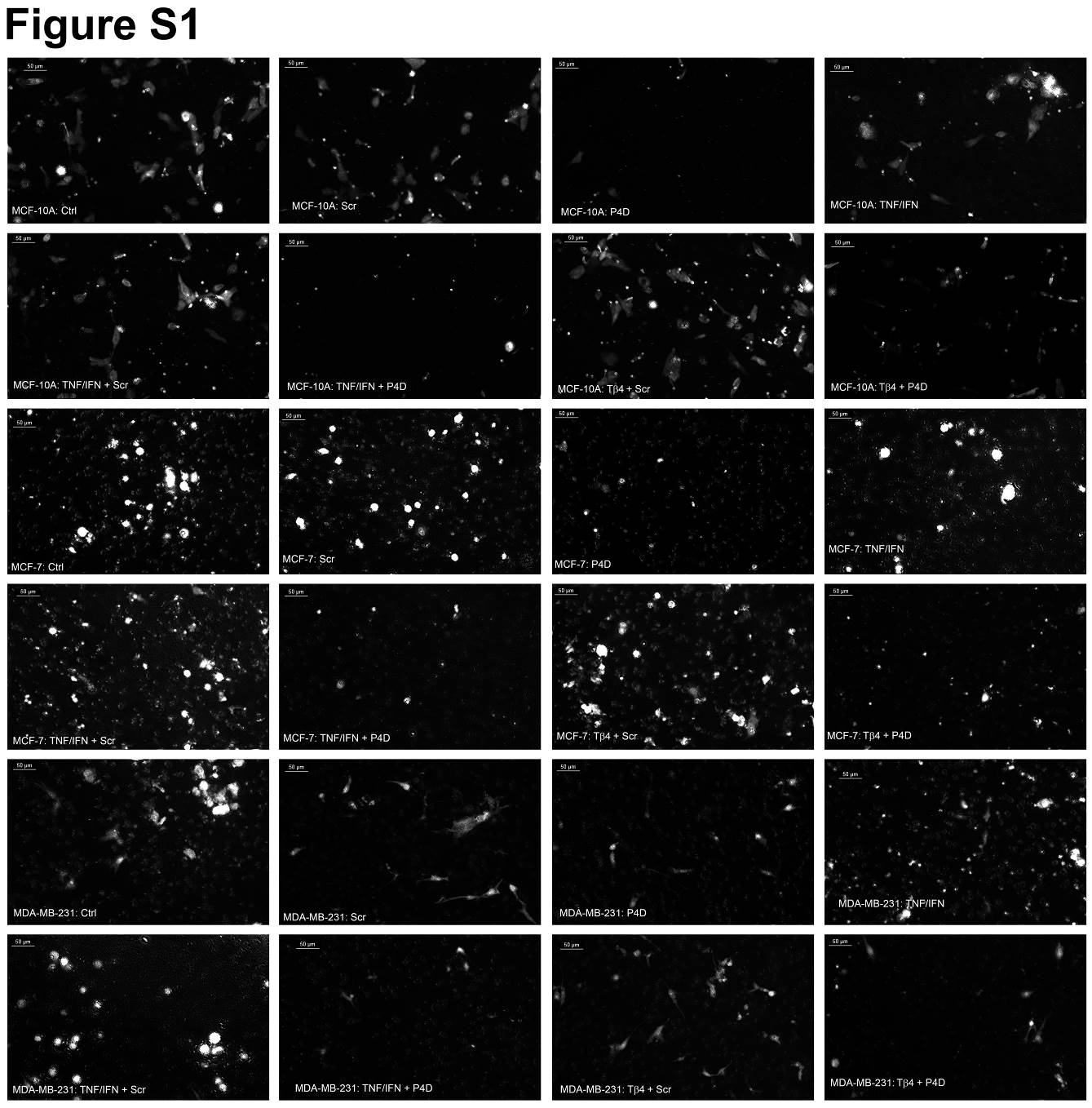

Supplement: Supplementary file 1 — Supplementary material 1 (DOC 216 kb) [file 10549_2019_5471_MOESM1_ESM.doc]
